# The effect of clinician-patient alliance and communication on treatment adherence in mental health care: a systematic review

**DOI:** 10.1186/1471-244X-12-87

**Published:** 2012-07-24

**Authors:** Laura Thompson, Rose McCabe

**Affiliations:** 1 Unit for Social and Community Psychiatry, Barts & the London School of Medicine and Dentistry, Queen Mary University of London, Newham Centre for Mental Health, London, E13 8SP, UK

**Keywords:** Communication, Alliance, Adherence, Mental health

## Abstract

**Background:**

Nonadherence to mental health treatment incurs clinical and economic burdens. The clinician-patient alliance, negotiated through clinical interaction, presents a critical intervention point. Recent medical reviews of communication and adherence behaviour exclude studies with psychiatric samples. The following examines the impact of clinician-patient alliance and communication on adherence in mental health, identifying the specific mechanisms that mobilise patient engagement.

**Methods:**

In December 2010, a systematic search was conducted in Pubmed, PsychInfo, Web of Science, Cochrane Library, Embase and Cinahl and yielded 6672 titles. A secondary hand search was performed in relevant journals, grey literature and reference.

**Results:**

23 studies met the inclusion criteria for the review. The methodological quality overall was moderate. 17 studies reported positive associations with adherence, only four of which employed intervention designs. 10 studies examined the association between clinician-patient alliance and adherence. Subjective ratings of clinical communication styles and messages were assessed in 12 studies. 1 study examined the association between objectively rated communication and adherence. Meta-analysis was not possible due to heterogeneity of methods. Findings were presented as a narrative synthesis.

**Conclusions:**

Clinician-patient alliance and communication are associated with more favourable patient adherence. Further research of observer rated communication would better facilitate the application of findings in clinical practice. Establishing agreement on the tasks of treatment, utilising collaborative styles of communication and discussion of treatment specifics may be important for clinicians in promoting cooperation with regimens. These findings align with those in health communication. However, the benefits of shared decision making for adherence in mental health are less conclusive than in general medicine.

## Background

Clinical practice in mental health has transformed in recent decades, principally fuelled by pharmacological advances. Consequently, the composition and objectives of psychiatric encounters have changed and in turn the roles of clinicians. The development of atypical antipsychotics and successful use of Selective Serotonin Reuptake Inhibitors render psychopharmacology an increasingly primary task [1]: A central function of consultations is medication management via regular review and regime modification [2]. This shift in role definition raises questions about the purpose and nature of the clinician-patient relationship and its influence on salient outcomes of the process of care including adherence to prescribed treatment e.g. what must a clinician say and do to optimise patient engagement? Adherence is defined as the extent to which the patient’s behaviour coincides with medical or health advice [3] and constitutes a crucial intermediate outcome for most mental disorders. Deviation from prescribed regimens may incur clinical and economic burdens including relapse, rehospitalisation and poor prognosis. Determinants of adherence span several factors relating to demographics, illness, attitudes towards treatment and psychosocial issues [4]. Side effects also present a consistent and understandable challenge for patients. Discontinuation of assigned treatment may owe to inefficacy or intolerable effects of medication [5]. In terms of a potential point of intervention, it is important to fully understand the role of the clinician-patient relationship, or ‘alliance’ as it will be referred to hereafter. ‘Compliance therapies’ have been trialled by clinicians, based on cognitive behavioural techniques. Trained clinicians deliver specific therapeutic interventions that topicalise medication cessation and relapse, normalising rationales for stigma and recognition of characteristic prodromal symptoms [6]. These appear to retain benefits in the short term [7]. However, standard psychiatric encounters involve non-specific counselling to stimulate positive attitudes towards treatment, found to be equally as effective in the longer term [8]. Central to achieving a beneficial alliance in this context is clinician-patient communication. A link between communication and patient adherence has been observed extensively in general medicine. A recent meta analysis synthesising results from correlational and experimental studies found the odds of a patient adhering to be 2.16 times greater if their doctor is a good communicator [9]. Despite this, such reviews of communication typically exclude studies of psychiatric patients [9,10].

‘Good’ communication is commonly expounded through a ‘patient centred’ model in which principles of patient involvement and collaboration are advocated [47]. Collaborative communication and inclusion of the patient’s perspective in relation to treatment decisions specifically i.e. Shared decision making (SDM) has emerged as a pivotal component in policy for mental health [11]. Deemed ethically laudable and found to yield improvements on outcomes in physical health [12], both parties are encouraged to take steps to reach consensus about treatment, engaging in patient-centric communication that accounts for individual preferences. This is based on the expectation that it will increase self-determination and in turn patient treatment adherence [13]. Little research has however systematically examined the impact of SDM on outcomes in mental health to affirm this [13,14]. Moreover, application of such concepts is hampered by a lack of clear definition and measurement, rendering the specific behaviours and communication practices underlying patient centred care and engagement unclear. No review to date has collectively examined alliance and communication in mental health in order to determine the aspects of each phenomenon that mobilise adherence behaviours.

This article presents an integration of evidence about the empirical grounding for relationship variables, behaviors and messages instrumental to promoting engagement with mental health treatment. The primary objective was to identify whether an association exists between clinician-patient alliance or communication and treatment adherence in mental health care. Secondary objectives were to locate specific aspects of the therapeutic encounter that may be harnessed to improve treatment and describe the characteristics of literature. Whilst alliance and communication are interlinked they represent distinct phenomena. Alliance is a subjective psychological construct held by participants on each another and their interaction [15] e.g. bond, goals, rapport or agreement [16]. Communication refers to components of the behavioural exchange between clinician and patient [15] with the potential to described either subjectively and objectively e.g. information giving and collaboration [17]. Associations with adherence are differentiated in this review accordingly. The term alliance is broadly used to represent the clinician-patient relationship. However, it may be construed elsewhere as the ‘therapeutic relationship’, ‘therapeutic alliance’, ‘helping alliance’ or ‘working alliance’ [18].

## Methods

### Search strategy

A rigorous journal screening was undertaken including all electronically registered references up to December 2010. An additional hand search in key journals of relevant professional categories, grey literature and dissertations was also performed. Table 1 denotes the sources used.

**Table 1:** Search resources

**Databases**	**Hand search**	**Grey literature**
Pubmed	The British Journal of Psychiatry	System for Information on Grey Literature (SIGLE)
PsycInfo	The American Journal of Psychiatry	British National Bibliography for Report Literature
Web of Science	Schizophrenia Bulletin	British Library Direct
Cochrane Library	Archives of General Psychiatry	Proquest Digital Dissertations
Embase	Acta Psychiatrica Scandinavica	
CINAHL.	Journal of Psychiatric & Mental Health Nursing	
	Journal of Mental Health	
	Journal of Advanced Nursing	
	Issues in Mental Health Nursing	
	Journal of Psychosocial Nursing	
	Health and Social Care in the Community	
	British Journal of Occupational Therapy	
	Canadian Journal of Occupational Therapy	
	American Journal of Occupational Therapy	
	Journal of Occupational Science	
	British Journal of Social Work	
	Social Work in Mental Health	
	Journal of Social Work Practice.	

Table 2 depicts the terms used in the search process, how they were combined and where truncation was used in order to capture all relevant variants of the terminology. Terms were categorised in four groups based on the research question, representing synonyms or specifications of; ‘communication’ or ‘alliance’ (group 1) between patients diagnosed with ‘mental disorder’ (group 2) and ‘professionals’ (group 3) and its impact on ‘adherence’ (group 4). In databases where limits were imposed on search terms, the key terms i.e. communication/alliance and adherence were used. The search process was augmented by personal correspondence with experts, advising on appropriate terms and relevant literature.

**Table 2:** Search terms with truncation

**Group terms combined by ‘AND’**			
**combined by “OR”**	**combined by “OR”**	**combined by “OR”**	**combined by “OR”**
**→ group term 1**	**→ group term 2**	**→ group term 3**	**→ group term 4**
**Group 1**	**Group 2**	**Group 3**	**Group 4**
communicat*	psychosis	psychiatr*	adher*
talk	psychotic	Doctor	complian*
interact*	schizophr*	mental health nurs*	concordan*
expressed emotion	schizoaffective	psychiatric nurs*	nonadher*
conversat*	delusional	social work*	noncomplian*
discourse	depress*	psycholog*	concordan*
dialogue	dysthymi*	care coordinator	persistence
relationship	bipolar	counsel*	treatment usage
alliance	cyclothymi*	therapist	attendance
shared decision making	panic	support work*	engag*
	agoraphobia	Psychosocial intervention work*	rejection of therapy
	phobia	employment coach	DNA
	obsessive compulsive disorder	nurse practitioner	drop out
	post traumatic stress disorder	case manager	medication possession ratio
	stress	vocational rehab specialist	service use
	anxiety	psych tech	
		physician	
		provider	
		practitioner	

### Inclusion and exclusion criteria

Prior to the screening, strict inclusion criteria were specified to orient the search filtering process. For studies to be deemed relevant, they included patient (or professional-patient) samples where participants were aged 18–65 and receiving treatment for psychotic disorders, anxiety disorders or mood disorders. The search was constrained to the main clinical disorders (Axis 1) according to DSM 1 V [19] to limit heterogeneity. Professionals were defined as any clinician in contact with this group within inpatient, community, primary care or outpatient settings. Pertinent data was that which had been collected via a subjective rating of the clinician-patient alliance/interaction using a validated scale or where a clear description of how the variable had been measured was provided e.g. via a single item assessing the state of the relationship [32]. Alternatively, an objective record and assessment of naturally occurring communication was necessary (e.g. with ratings by independent researchers of audio or video taped recording). Also required, was an assessment of patient adherence, via direct (e.g. pill count, blood test) or indirect (e.g. patient or clinician self-report) measures. ‘Adherence’ in this review pertained to medication-taking behaviour and/or appointment attendance. The resulting analysis was considered appropriate if it, at minimum, assessed the relationship (correlation) between alliance/communication and adherence, or tested for a significant difference between adherers and nonadherers in relation to these variables. All included studies were in compliance with the ethical principles of the Helsinki declaration and approved by appropriate bodies.

### Screening

**Figure 1:**
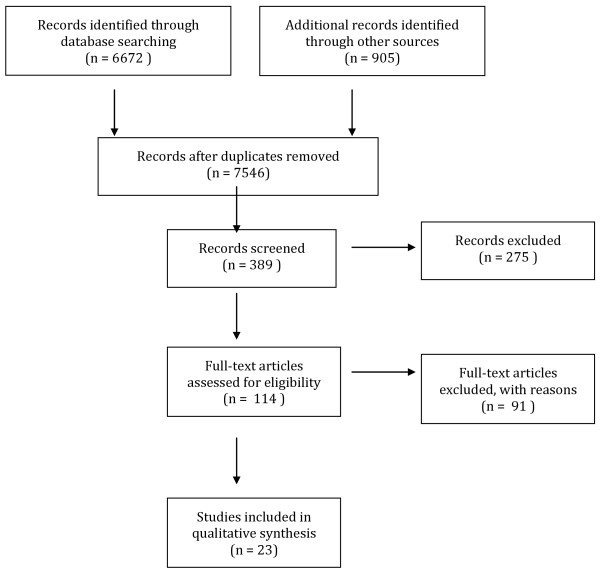
Quorum diagram.

Following searching, the resulting titles from each database were screened in accordance with the research aims. Potentially relevant abstracts were obtained for further examination, a random selection of which (20%) were screened independently by a secondary researcher to check reliability and minimise potential bias. Full texts of the selected abstracts were then retrieved for more rigorous inspection and application of the exclusion criteria. 6672 titles were found of which 6283 abstracts were considered irrelevant, leaving a total of 389 abstracts for screening after the removal of 31 duplicates. Following further filtering, 114 full texts were examined in their entirety of which 20 met the review’s inclusion criteria. Whilst review articles were automatically excluded in the search, reference sections were inspected for potentially applicable citations. Three studies were also retrieved from this additional search process. Reasons for exclusion were recorded and discrepancies discussed between independent researchers until consensus was met. Figure 1 outlines the results of the screening protocol using Quorum guidelines [20]. Only studies fulfilling all inclusion criteria were reported in the final review (n = 23).

### Data extraction

Data extraction was conducted using an instrument designed for this review to elicit all relevant aspects of included articles. This allowed for both qualitative and quantitative accounting of the study including author, title, year, country, treatment setting sample, aims, data source, measures used, analysis, results, specific alliance/communication variables associated with adherence, and limitations of the study. Whilst numerous articles referred to general predictors of adherence, only outcomes relating to alliance or communication were extracted. Two reviewers independently recorded the data, consulting with a third reviewer in the event of disparate documentation of study features.

### Quality assessment

Quality assessment (QA) of the included studies was problematic as there is no ‘gold standard’ design for studies of clinician–patient alliance or interaction. A review of QA for non-randomised trials demonstrated six tools applicable to systematic reviews [60], from which Down’s et al. (1998) [21] was selected as a guide for its comprehensiveness. The studies were assessed on four dimensions; reporting, external vali-dity, internal validity and power. The tool required adaptation to make it more applicable to the nature of papers reviewed. For example, as the majority of studies (n = 18) had no ‘interventions’, control groups or blinding, questions pertaining to these issues were removed. Instead, an additional QA variable was added to assess the study design and it’s potential to allow for causal hypotheses. Overall, study ratings for each category were denoted as percentage score. The QA criteria are outlined below.

1) Reporting: Do studies provide a clear description of aims, outcomes, characteristics of patients, findings and actual probability values? (Total/5).

2) External validity: Are those patients asked to participate in the study were represented of the entire population from which they were recruited? Patients would be representative if they consisted of the entire source population, an unselected sample of consecutive patients, or a random sample (Total/1).

3) Internal validity: Are the statistical tests used to measure the outcomes appropriate? Are both adherence and alliance/communication measures validated and reliable? Was there adequate adjustment for confounding in the analyses from which the findings were drawn? (Total/3).

4) Study design: To what extent can the study identify causality? Scores differentiate between cross sectional, prospective/longitudinal and experimental designs (Total/2).

Overall Table 3 suggests a moderate quality of evidence in this field. Of the 23 studies, 5 had low quality scores (≤ 50%) 12 had moderate scores (>50%) and 6 obtained high scores (≥ 70%) according to the determined threshold for high quality [21]. All studies exhibited a strong standard of reporting and used appropriate statistical tests. However, a number of collective limitations can be identified. Validated measures were not consistently implemented and external validity was low. Often random sampling or consecutive admissions were not used to recruit participants or detail was often insufficient to establish whether those who agreed to participate were representative of the entire source population. All studies also incurred weak ratings in relation to power due to a lack of formal sample size calculation. Furthermore, identifying directionality of effect was problematic in the majority of research reviewed. Most studies adopted cross sectional designs and only four studies examined the effect of communication on adherence experimentally. The complexity of adherence determinants are well documented, but studies inadequately captured and discussed confounding in the analyses. For example, most studies failed to capture and adjust for side effects, consistently identified as one of the most significant reasons for drug treatment failure across a range of mental health disorders [4,43].

**Table 3:** Quality assessment ratings for included studies

**Paper**	**Reporting**	**External validity**	**Internal validity**	**Power**	**Study design**	**Study quality score %**
**Sajatovic et al. (2006)**[22]	5/5	0/1	1/3	0/1	0/2	50%
**Weiss et al. (2002)**[23]	5/5	1/1	2/3	0/1	1/2	75%
**Corriss et al. (1999)**[24]	4/5	1/1	2/3	0/1	1/2	67%
**Lecomte et al. (2008)**[25]	5/5	0/1	2/3	0/1	0/2	58%
**Startup et al. (2006)**[26]	5/5	0/1	2/3	0/1	0/2	58%
**Olfson et al. (2000)**[27]	5/5	1/1	1/3	0/1	1/2	67%
**Frank et al. (1990)**[28]	5/5	0/1	2/3	0/1	1/2	67%
**Zeber et al. (2008)**[29]	5/5	0/1	2/3	0/1	0/2	58%
**Perron et al. (2009)**[30]	5/5	0/1	2/3	0/1	0/2	58%
**Madsen et al. (2009)**[31]	4/5	0/1	2/3	0/1	1/2	58%
**Shigemura (2010)**[32]	5/5	0/1	1/3	0/1	0/2	50%
**Yeh et al. (2008)**[33]	5/5	0/1	1/3	0/1	0/2	50%
**Bull et al. (2011)**[34]	5/5	0/1	1/3	0/1	0/2	50%
**Lin et al. (1995)**[35]	5/5	0/1	1/3	0/1	1/2	58%
**Gonzalez et al. (2004)**[36]	5/5	0/1	2/3	0/1	1/2	67%
**Bultman et al. (2000)**[17]	5/5	0/1	1/3	0/1	1/2	58%
**Mahone et al. (2008)**[37]	4/5	0/1	2/3	0/1	0/2	50%
**Hamann et al. (2006**[14]	5/5	0/1	2/3	0/1	2/2	75%
**Loh et al. (2007)**[38]	5/5	0/1	2/3	0/1	2/2	75%
**Loh et al. (2007)**[39]	5/5	0/1	2/3	0/1	2/2	75%
**Ludmen et al. (2003)**[45]						
**Von Korff et al. (2003)**[41]	5/5	0/1	2/3	0/1	2/2	75%
**Sleath et al. (2003)**[42]	5/5	0/1	2/3	0/1	1/2	67%

### Analysis and findings

Criteria for conducting meta-analysis were not fulfilled due to variability in the statistical procedures and measures used to analyse relationship, communication and adherence outcomes. The findings of the systematic review are therefore synthesised below in accordance with the original research questions. Seventeen studies reported positive associations with adherence. Results were considered positive if study authors reported a statistically significant (p < 0.05) association between adherence and at least one relationship/communication variable, or a statistically significant difference between adherent and non-adherent patients on such measures.

The majority of studies focussed on patients diagnosed with depression (n = 16). The remaining studies investigated participants with a psychotic disorder (n = 8) or, least frequently, bipolar disorder (n = 3). Literature was conceptualised as three categories on the basis that alliance and communication may differentially influence adherence: Group 1: global measures of clinician-patient alliance (n = 10). Group 2: specific communication styles and/or or messages communicated by the treating clinician (demonstrated via subjective measures or experimental interventions) (n = 6). Group 3: independently coded recordings of naturally occurring clinical communication (n = 1). Discussion of the studies and their pertinent results are reported within the proceeding narrative synthesis.

## Results

### Clinician- patient alliance and treatment adherence

Alliance is consistently associated with adherence in mental health. Of 10 studies (2 low quality, 7 moderate and 1 high), eight yielded significant associations to some aspect of the clinician-patient relationship. Table 4 (See Additional File 1: Table S4) outlines the characteristics and findings of all included papers. The clinician patient alliance was most frequently assessed via the Working Alliance Inventory (WAI) [16] (n = 4). This instrument, used in patient/clinician self-report or observer rated versions, provides measures of three related components hypothesised to determine the degree and quality of clinician helping alliances: a) patient and therapist agreement on the goals of treatment, b) patient and therapist agreement on the tasks to achieve these goals and c) the development of a personal bond between patient and therapist. Whilst two studies found no positive associations when analysing these factors globally [22,25], one study combining cross sectional and longitudinal prospective analyses found adherence difficulties not only to be associated with weaker working alliance, but demonstrated its impact on the time-course of adherence maintenance in initially adherent patients and the development of adherence in initially nonadherent patients [23].

A particularly important aspect of the working alliance may be patient-clinician agreement on the tasks of treatment, found by two studies to be significantly associated with patient adherence in subscale analyses [24,26]. In a psychotherapy (Cognitive Behaviour Therapy) setting, therapist and patient agreement on the goals of treatment was also relevant to premature termination of therapy [26]. However, arguably this finding may be due to the formulaic nature of the therapy where clearly defined goals are inherent to the process. Mainstream psychiatric services differ from conventional psychotherapy in important respects including the commonly open-ended nature of treatment, actual or potential use of coercive treatment measures and higher variability of the frequency, length and goals of consultations [15]. In attempt to address this issue the same study used a dual measure of adherence, the Active Engagement Scale, a 15-item questionnaire, assessing primarily involvement in therapy and collaborative participation. Three studies [26-28] found significant associations with this definition of alliance. As prospectively observed over a two year period, the more actively engaged in therapy patients become the more likely they may be to take their medication as prescribed [28]. Moreover, particularly crucial dimensions of active engagement for adherence may be patient optimism about the usefulness of treatment, meaningful involvement in therapy, patient interest in understanding their illness and realistic perceptions of the therapist [27].

The ‘alliance’ has also been extended beyond the primary treating clinician and patient to encompass entire mental health teams. Two studies [29,30] solicited perceptions of the therapeutic alliance using The Health Care Climate Questionnaire (HCCQ) [40]. This 10-item bipolar disorder-specific instrument, elicits the degree of comfort a patient expresses with their treatment according to statements regarding their care environment as it has been developed by their mental health team. For example, ‘I feel encouraged by my mental health team’ and ‘I am encouraged to ask questions about my treatment’. One study analysing the overall HCCQ rating found a significant association with the baseline measure of alliance but not 1 year follow up [30]. This suggests that provider team support may contribute to client motivation and engagement. However, the causal associations are unclear. In the second study, adherence outcome was differentiated by missed doses and interpersonal barriers to adherence. The Overall HCCQ score was significantly associated with interpersonal barriers to adherence but not missed medication doses. Similarly, in individual item analyses, certain alliance variables were associated differentially to the two dimensions of adherence measured. Salient aspects of team alliance related to both measures were identified as conveying confidence in the patients ability to make changes, ensuring the patient and team stay in frequent contact and regularly reviewing the patient’s progress in managing all aspects of their treatment plan. Finally, in a significantly larger sample, an internet-based survey among 1151 patients with major depressive disorder found low adherence was associated with, and predicted by, a neutral or negative doctor-patient relationship [32].

### Communication styles and clinical messages related to adherence

Other studies have employed greater specificity in capturing aspects of the clinician-patient interaction. This enables identification of candidate communication styles and clinical messages that may mobilise adherence. Of 12 studies (3 low quality, 4 moderate and 5 high), eight found at least one or more variable to be postively associated. So how should clinicians co-act with patients to optimise the therapeutic encounter? The extent to which patients perceive behavioural traits such as listening, empathy and respectfulness to manifest in the clinician’s communication style relates to their following of medication directions [33]. However, the practitioner’s ability to elicit patient’s perspective about treatment may be specifically important. Patients who identify a participatory decision making style in clinicians, their propensity to involve them in treatment decisions, have been found to be more adherent at 6 months follow up [36] and 6–8 weeks post initial consultation [38]. Imparting information within such an approach, including facts about the disease and treatment, may also be factorial in adherence behaviours [38]. Evidence for SDM is however inconsistent. In another sample, the degree to which outpatients viewed their prescribing doctor as exhibiting a collaborative style in relation to similar items was not associated with subsequent adherence at 3, 6, 9 or 12 weeks [31]. Whilst an explanatory hypothesis for contradictory findings could be variation in patient preference for decision involvement, a further study found no association between perceived participation and adherence, even when the degree of patient involvement was aligned with their preference for SDM [37].

These studies examined subjective perceptions of SDM. However, the construct has also been explored experimentally with similarly inconsistent findings. Three trials explored the impact of a multi faceted intervention. The first applied a decision aid, a 16 page booklet addressing the pros and cons of alternative types of antipsychotic medication, and a planning talk between patient and physician to establish agreement on further treatment [44] In the second, physicians were trained in SDM and a decision board for use during encounters was distributed to the patients, in addition to evidence based information about depression care and specific encouragement for patients to be active in the decision making process [39]. Though such tools are designed to enhance patient involvement, both studies found no effect on adherence compared to routine care. In the third sample however, clinicians were trained to provide a complex intervention involving an SDM element. Intervention patients received an educational book, videotape about effective management of chronic or recurrent depression, in-person visits and telephone monitoring and were significantly more likely to refill antidepressant medication prescriptions than usual care patients during the one year follow up period [41,45].

If indeed collaborative communication stimulates adherence behaviours, its positive influence may be attributable to an intervening variable i.e. enhancing patient beliefs about the medication prescribed [17]. One unique study tested a theoretical model suggesting that physician (initial and follow up) collaboration style influences client medication beliefs and in turn medication-taking behaviour. Fundamental elements of physician initial communication style, derived form the Health Communication Model [46] included the degree of friendliness during the visit, asking if the patient had questions or concerns, assisting with issues relating to the use of medication, providing clear instructions on how to take medication, clearly explaining how the antidepressant would affect the patient and talking about actions the patient can take to feel better. Key components of the follow up communication style were considered to be the extent to which the physician encourages expression of concerns or problems with taking medication, asks about and listens to concerns about medication and helps solve problems related to the patient’s use of medication. This study highlights the importance of collaborative communication about treatment specifics more generally rather than specifically treatment decisions. Consistent with this, two studies [34,35] identified distinct medication-related messages that may have a bearing on adherence behaviour. That is, the time period patients are instructed to take their medication and discussion regarding side effects may be instrumental in decreasing the odds of discontinuing antidepressant therapy. Patients told to take their medication for longer were found to be less likely to adhere to their medication. Furthermore, communication regarding adverse effects may significantly decrease the odds of terminating antidepressant therapy [34].

Potential medication-related motivators of adherence in the initial phase of antidepressant treatment have also been identified [35]. These include physician question asking about prior use of antidepressants, instructions provided by the doctor i.e. to continue medication use even when symptoms have alleviated; check before discontinuing medication; take medication daily, and advising the patient what to do in case of questions. General discussion of pleasant activities may also be important to initial motivation to adhere to regimens. In this study, no communication variables were found to be significant predictors of later stage adherence, suggesting important topics are be dependent on the stage of treatment. Another study assessing the same communication messages did not find them to be related to antidepressant adherence [36].

### Objectively measured communication and adherence

Communication signifies an observable behavioural exchange between the patient and clinician and therefore has the potential to be captured in objective terms by an independent observer [15]. Despite this, only one study (of moderate quality) examined the relationship to adherence via this method [42]. Analysis was performed on a data set including 27 resident physicians who were each audio taped with 6 to 21 of their Spanish or English speaking patients within general internal medicine or family practice. Researchers coded specific communication variables from transcripts including 1) discussion of antidepressants during the encounter 2) number of different types of information the doctor provided the patients about antidepressants 3) number of physician questions about antidepressants 4) number of different types of information the patient stated regarding antidepressants 5) number of questions the patient asked about antidepressants. Adherence was assessed for the 100-day period after the audio taped consultation via pharmacy prescription refill records. Analyses determining how the communication variables were related to adherence, demonstrated that only patient question asking was significant. That is, patients who asked more questions regarding their medication during the encounter were less adherent to their therapy during the 100-day period after their visit. However the fact that medication-specific discussion was not found to be associated with adherence is in contrast to the previous research reviewed.

### Variability in adherence measures

The variability in assessment and definition of adherence, as evident in Table 4, presents an important consideration as comparability between studies is affected. Most frequently, adherence was assessed via patient report (n = 14), consistent with an identified preference for this method in mental health [48]. Choice of measures and criteria for non adherence however were heterogeneous, ranging from patients being asked if they had stopped taking their medication for a period of one week or more during the intermittent follow up period [27], to patient’s rating on a scale of 0–100 how often they forgot to take their medication, alter their dose, or miss a dose intentionally to suit their needs. The only measure to be duplicated between studies [29,30] was the Morisky scale [49]. Three studies used a combination of therapist and patient reports [28,39,44] and two studies [23,24] used solely therapist report, both of which employed a 4-point likert scale with ratings from 1 ‘active compliance’ to 5 ‘passive compliance’ [50]. Direct,i.e. objective, assessments were only used in four studies [26,36,42,44] in the form of pharmacy refill records, premature termination of therapy and blood plasma levels. Subjective measures were therefore predominantly adopted which, particularly when used in isolation, can be susceptible to bias. For example, exaggerating the degree of adherence (patient self-report) and basing adherence judgements on deteriorating clinical state or inaccurate perception of agreement about treatment [51] (therapist-report). In line with existing literature [48], this review highlights the opportunity for advancement in adherence research. Most notably in relation to consensus development, allowing for studies to be compared on a common variable. In turn, more valid conclusions could be drawn when assessing the effect of alliance or communication.

## Discussion

### Summary of main results

To our knowledge, this is the first review to examine alliance, communication and adherence in mental health. Twenty-three articles met the inclusion criteria. The methodological quality overall was moderate, reflecting largely cross-sectional nature of the research in this field and limitations in comparability and identifying causality. Ten papers examined the relationship between adherence and the clinician-patient alliance, which emerged as a consistent predictor, though its components have differential significance. Twelve studies examined specific communicative styles or messages, only four of which employed intervention designs. Shared decision making yields inconsistent results in relation to patient engagement. However, collaborative features of communication more generally, such as imparting medication-related information and discussing the practicalities of treatment specifics, were positively associated, though less studied. Only one study explored the association between adherence and observer rated clinical communication, highlighting a gap in psychiatric literature for more objective methods of communication measurement. Specific mechanisms related to treatment engagement are discussed in more detail.

### Specific communication mechanisms that result in patient engagement

It is commonplace to conceptualise the clinician-patient relationship as one depending on ‘bond’ and rapport [52,53]. This review shows that in relation to adherence, more task-oriented elements of the alliance may be instrumental. Agreement on the tasks of treatment, collaborative participation and regularity of contact with clinicians for example emerge as ‘active’ elements of the alliance. The treatment context may however influence the magnitude of benefit from these factors e.g. Goal elements in a psychotherapy setting [26] may preside over such task-based features relative to an outpatients setting [22]. It is unclear how a positive clinician-patient alliance translates to specific communication styles and messages that can be utilised to improve engagement. This is attributable to less consistent findings in the field of communication research and a lack of observed naturally occurring clinical communication. However, this review presents two main candidates for clinicians to consider. SDM provides a model of communication to enhance patient involvement in the decision process of consultations, but is inconsistently associated with adherence in mental health. Whilst associated tools such as decision aids help patients make deliberative choices among treatment options and a provide a platform from which they can assess risks and benefits, non-significant associations with adherence outnumber positive. This uncertainty is further complicated by difficulties in examining effects of complex interventions with SDM elements. For example, arguably the positive outcomes in Ludmen/Von Korff et al. [41,45] cannot be ascribed solely to the SDM element as it was part of a multi-faceted intervention. Sharing preferences about treatment may be particularly challenging in mental health care due to the nature of symptoms that make establishing a shared understanding about treatment problematic. Research must identify the complications of sharing decisions in these contexts to further understand the relationship with adherence behaviours.

Despite this, collaborative communication throughout the consultation more generally appears vital. Clinicians who are friendly, explain medication, address questions and concerns and discuss treatment specifics e.g. medication instructions, are more likely to have patients who adhere to regimens. Though further pathway research is necessary to reinforce findings [54], the mechanism by which this occurs may be enhancement of patient beliefs about medication. The emphasis on medication-specific discussion certainly aligns with the notion that knowledge maybe an important patient factor clinicians can influence in order to improve adherence in mental health. Patients need to be informed about and comprehend treatment. Coupled with the finding that clinician optimism is associated with adherence, perhaps provider attitudes towards treatment, manifest in communication, can influence patient’s expectations of prescribed treatment. Indeed, when studied outside of the context communication, clinician attitudes towards medications have been found to impact patient’s medication adherence in mental health [55].

Only one study was able to objectively identify a specific communication practice, patient question asking, as related to adherence. Given the finding that communication may mediate beliefs and knowledge about medication [17], perhaps more question asking reflects less understanding on the part of the patient. Further studies of this nature may enable identification of specific practices that either indicate patients risk of nonadherence, or are involved in mobilising self care behaviours in mental health. This review has demonstrated a clear paucity of objectively measured natural clinical communication in mental health.

### Comparison to literature

The findings of this review are consistent with mental health research identifying the alliance to be associated with other treatment outcomes [56]. An emphasis on agreement about tasks of treatment is in line with perceived patient agreement being associated with adherence in general medicine [51]. It has been suggested that discussing treatment specifics, highlighted in this review, enhances clinician’s ability to perceive such patient agreement [51]. The potential for perceived collaboration in clinical encounters to encourage patient engagement aligns with findings in general medicine that collaboration is associated with improved adherence [57]. Inconclusive findings in relation to collaborative treatment decisions specifically i.e. SDM is inconsistent with more positive outcomes in general medicine [12], but consistent with reviews in mental health examining SDM and a range of patient outcomes [13]. Interestingly, unlike general medicine where numerous empirical studies from various populations and settings link systematically coded communication to adherence [9], only one study objectively measured naturally occurring clinical interactions, suggesting a research deficit in psychiatry specfically.

### Limitations

Whilst informative, the review should be considered within the context of its limitations. No meta-analysis was conducted due to the heterogeneity of methods. Estimates of overall effects could not therefore be calculated. It also remains possible that studies in this field are susceptible to publication bias hence the frequency of positive associations relative to negative ones may be overstated. Furthermore, papers in mental health were examined collectively rather than differentiated by disorder. Arguably, this distinction could be important if settings i.e. inpatient/outpatient or diagnostic groups have different communicative needs. Clinician-patient alliance and communication in schizophrenia for example, addressed in 7 studies, is complicated by the nature of psychotic symptoms [58].

### Implications of findings for future research

This review underlines methodological weaknesses that may be useful to address in future research. Only six studies were deemed high quality according to QA criteria. Whilst practical constraints of cross-sectional studies in naturalistic settings are expected, general improvements to derive from these findings relate to; increasing implementation of validated measures and supplying adequate information on reliability and internal consistency; striving for larger sample sizes and performing formal sample size calculations to increase precision in extrapolating effects to the wider mental health care population; employing random sampling or recruiting consecutive patient admissions to optimise external validity and the potential inferences that can be made.

Beyond this, five areas may offer particularly salient improvements to research in this field; Firstly, whilst alliance was mostly assessed via validated instruments, the dimensions of this construct varied between measures. It may be important to distinguish between components in analyses and explore their differential associations with adherence, e.g. clinician-patient agreement about the tasks of treatment may be more important than bond, in order to enhance the potential application of findings in clinical practice and training. This may be possible by performing subscale analyses of measures in addition to global ratings.

Secondly, whilst alliance and communication are interrelated, they are analytically distinct concepts. It is therefore difficult to consider how specific communication practices are involved in the formation of a positive alliance. Furthermore, generalised communication coding categories such as ‘discussions about medication’ may fail to account for the wide range of behaviors and content these interactional events may incorporate. Investing more effort, and specificity, in studying clinical communication variables may provide a fruitful starting point from which we can look at how, and if, such practices are related to subjective perceptions of alliance and outcomes like adherence. More objective micro analytic methods such as conversation analysis that account for context, sense making and clinician-patient interactivity may be useful approaches in achieving this.

Thirdly, whilst it is appealing to conclude that alliance and communication have an impact on patient cooperation with treatment regimens, it is important to contextualise any effect within the interplay of other possible adherence determinants e.g. illness related, demographic and psychosocial factors. These potential confounders should be sufficiently adjusted for in future research so valid inferences about the effect of the clinical interaction can be made.

Fourthly, longitudinal prospective studies that follow patients and clinical interactions over time are necessary to account for the potentially dynamic nature of adherence behavior and that the clinician-patient alliance may change or develop. To increase comparability between studies it may be useful for each investigation to report a mean percentage of medication consumed throughout the follow up period, even if the primary measure of adherence is operationalised otherwise [48].

Finally, due to the largely correlational nature of research reviewed, the directionality of effect can only be speculated upon. The question of whether the predictive power of the alliance or communication derives from the effect of patient engagement itself on the interaction, or even patient characteristics present at the start of treatment, remains pertinent in future research. Further experimental studies or use of causal modelling techniques would allow researchers to elicit directions of causality and eliminate the alternative explanation that a strong alliance is not a pre-requisite for better adherence, but rather a consequence of positive clinical change.

## Conclusions

Adherence to mental heath treatment is frequently a challenge for practicing clinicians. Treatment and patient factors have attracted most attention in published research. However, this review shows providers play a role in mitigating nonadherence. A positive alliance is associated with more favorable adherence. How this translates into tangible communication practices, and the mechanisms by which these may influence treatment engagement are less conclusive and require more sophisticated studies and methodological techniques. Communication represents an observable exchange between patient and clinician that may be objectively described. However, adherence research relies mainly on subjective measures, making actual communication relatively under researched in mental health compared to medicine more widely. Addressing this deficit, and the considerable methodological limitations in this field, could facilitate better application of findings in clinical practice. Currently, literature implies providers should engage patients collaboratively in the consultation in order to establish agreement surrounding the tasks of treatment, an important aspect of alliance. Training clinicians to discuss treatment specifics, including patient concerns about treatment may improve their ability to perceive this agreement [51]; improve patient’s beliefs about and attitudes towards treatment; and gain insight into the idiosyncratic reasons outside of the clinical interaction also underling nonadherence. As such, whilst time constraints on psychiatric encounters pose a challenge to clinicians in developing bonds with patients, more effective collaboration on practical aspects of treatment may be one way of compensating for this. Clinicians should also observe features of patient communication e.g. question asking that maybe indicative of engagement both within, and external to, the consultation. SDM is outlined in policy as a preferred mode of communication that will improve patient adherence. However, further research is necessary to understand this relationship. Arguably, the implementation of such patient-centred principles may be particularly challenging in psychiatry [59]. Despite methodological deficiencies in this field, engagement in the psychiatric consultation itself may impact patient engagement with treatment more globally.

## Competing interests

The authors declare they have no competing interests.

## Authors’ contributions

LT and RM were involved in the conception of the review. LT designed the study, conducted the search, selected the studies, interpreted the data and drafted the manuscript.

## Pre-publication history

The pre-publication history for this paper can be accessed here:

http://www.biomedcentral.com/1471-244X/12/87/prepub

## Supplementary Material

Additional file 1 **Table S4.** Characteristics of included studies. Click here for file
