# Supplementary material for: The effect of clinician-patient alliance and communication on treatment adherence in mental health care: a systematic review
Source: BMC Psychiatry. 2012 Jul 24;12:87. doi: 10.1186/1471-244X-12-87 (PMC3528426; doi:10.1186/1471-244X-12-87)
Supplement: Additional file 1 — Table S4. Characteristics of included studies. [file 1471-244X-12-87-S1.pdf]

Table 4. Characteristics of Included Studies

Author	Sample & Setting	Study Design	Measure of Adherence & Rater	Measure of Alliance/Communication & Rater	Findings
**Sajatovic et. al (2006) [19]**	323 Veteran inpatients with a diagnosis of Bipolar disorder	Cross-sectional design	Patient self-report interview	The Working Alliance Inventory (WAI), patient & clinician version	No significant difference was found between full adherers and those reporting substansial nonadherence on WA scores from both patient and clinician report (p= 0.241)
**Weiss et. al (2002) [21]**	162 outpatients with schizophrenia (47.5%), schizoaffective disorder (45%) or other psychotic disorders (7.5%)	Cross-sectional and longitudinal prospective design	Therapist self-report	The Working Alliance Inventory, Short form, therapist version	Adherence difficulties were significantly associated with weaker WA (p=0.30). WA also predicted the length of time adherence was maintained (p <0.001) and the length of time it took patients to develop active adherence (p< 0.05)
**Corriss et. al (1999) [22]**	87 outpatients with schizoaffective disorder (48%) schizophrenia, paranoid type (38%), schizophrenia, undifferentiated type (12%) or affective disorder with psychotic features (2%)	Cross-sectional design	Therapist self-report	The Working Alliance Inventory, Short form, therapist version	Low agreement regarding the tasks of treatment were associated with with poor adherence (p<0.05) For adherence to non medication all three alliance variables were associated with poor adherence. (p < 0.05).
**Lecomte et. al (2008) [20]**	1118 patients from early intervention psychosis services with schizophrenia (56%), schizoaffective disorder (11%), bipolar disorder (10%) or psychosis NOS (10%)	Cross-sectional design	Patient self report- assessed with a combination of the MAS/MCS . Dosage frequency also assessed.	The Working Alliance Inventory, Patient version (Hovarth et al 1989)	Alliance did not emerge as a predictor of adherence (F= 1.22 (df 2,100) n.s.)
**Startup et. al (2006) [23]**	29 patients from a controlled CBT trial with schizophrenia (n=25), schizophreniform (n=4) or schizoaffective disorder (n=1)	Not stated (observational)	Premature termination of therapy (those who leave before session 12 considered 'drop outs', others stay ins'	Working Alliance Inventory, Observer version and the Active Engagement (AE) Scale	Stay ins were significantly more engaged with treatment as per the AE scale (p =0.01) and showed significantly more agreement on the goals (p=0.05) and tasks (p=0.04) of treatment, but did not differ significantly on the bond subscale (p= 0.08)
**Olfson et. al (2000) [26]**	213 inpatients with schizophrenia or schizoaffective disorder	Not stated (longitudinal prospective design)	Patient self-report interview at 3 month follow up post-discharge.	The Active Engagement Scale	Noncompliant patients received significantly poorer mean scores on 4/6 of the AES subscales: optimism about usefulness of treatment (p=0.006), meaningful involvement in therapy (p=0.09) interest in understanding their illness (p=0.015), realistic perceptions of the therapist (p=0.004).

**Frank et. al (1990) [25]**	143 hospital patients with schizophrenia	Not stated (longitudinal prospective)	Therapist reports, patient reports and medical records of psychotherapy utilisation and medication compliance for a two year period or until patients dropped out.	The Active Engagement Scale, therapist rated	The more actively engaged in therapy the patients became, the more likely they were to take their medication as prescribed (r= .37, df=70, p <.01)
**Zeber et. al (2008) [27]**	435 Veterans inpatient/outpatients with bipolar disorder, cyclothymia or shizoaffective disorder-bipolar subtype	Cross-sectional design	Patient self report: 1) patient report of no. of days within the past 4 dose has been missed. 2) Morisky scale:	Health Care Climate Questionnaire (HCCQ), patient rated	The overall HCCQ score demonstrated a significantly positive relationship with adherence outcome 1) ((OR 1.03, 95% CI 1.01-1.03, p=0.022) , but not 2) . For the individual HCCQ items 4 items were statistically significant in relation to outcome 1) and 5 in relation to outcome 2)
**Perron et. al (2009) [28]**	429 Veterans inpatient/outpatients with bipolar disorder, cyclothymia or shizoaffective disorder-bipolar subtype	Not stated<br><br>(Cross-sectional and longitudinal prospective design)	Patient Self reprt: Morisky Scale administered at baseline and 1 year-follow up.	Health Care Climate Questionnaire, patient rated	Medication adherence exhibited a significant association, but a small effect size with therapeutic alliance at baseline `(p=0.15, *p*=0.011), but not follow-up (p=0.02, *p*=0.799)
**Shigemura et. al (2010) [12]**	1151 individuals with major depressive disorder participating in an internet based survey	Not stated<br><br>(Cross sectional design)	Patient self-report	1 item patient-rated scale	Low adherence was associated with a neutral/negative doctor-patient relationship (P<0.001) and in a multivariate model low adherence was predicted by neutral/negative doctor-patient relationship (p=0.020)
**Yeh et al (2008) [31]**	401 outpatients with major depression or depressive disorder	Cross-sectional design	Patient self-report	Patient-rated questionnaire	The professional-patient interaction was a significantly correlated with medication adherence (p < 0.05), however did not emerge as a significant predictor of adherence.
**Bull et. al (2011) [42]**	181 outpatients with major depression or dysthymic disorder	Not stated<br><br>(Cross-sectional design)	Patient self-report	Patient & physician questionnaire	Patients who reported they were told to take their medication for less than 6 months had higher odds of discontinuing antidepressant therapy than patients who reported being told to take their medication for more than 6 months (OR 3.12 95% CI 1.21-8.07). Communication regarding adverse effects significantly decreased the odds discontinuing antidepressant (OR 0.49 95% CI 0.25-0.95).
**Lin et. al (1995) [43]**	155 primary care patients with major depression, minor depression or dysthymia	Obersvational cohort (longitudinal)	Patient self-report (early and late phase adherence – 1 & 3 months)	Patient rated questionnaire	Patients reporting having received more educational messages regarding medications and discussions of behavioural strategies were more likely to adhere to their antidepressants (p= 0.008.)

**Lin et. al (1995) [43]**	155 primary care patients with major depression, minor depression or dysthymia	Obersvational cohort (longitudinal)	Patient self-report (early and late phase adherence – 1 & 3 months)	Patient rated questionnaire	Patients reporting having received more educational messages regarding medications and discussions of behavioural strategies were more likely to adhere to their antidepressants (p= 0.008.)
**Gonzalez et. al (2004) [32]**	95 veteran outpatients with depression (or with comorbity) 2 groups: newly prescribed antidepressants or newly referred to clini	Prospective cohort study (longitudinal)	Short term adherence (1 month) assessed by pharmacy records (med group), chart notes (referral group). Intermediete adherence (6 months): medication possession ratio, chart notes	Patient rated participatory decision making style and patient-rated..questionnaire	Provider participatory decision making style was significantly associated with adherence at 6 months (p=0.03), but not 1 month in both groups. Those with a mental health referral reporting that their doctor had more characteristics of a participatory decision making style were more likely to attend their initial appointment (p=0.04)
**Bultman et. al (2000) [40]**	100 patients from community pharmacies receiving antidepressants.	Not stated (prospective observational)	Patient self report adapted from the Brief Medication Questionnaire (BMQ) at 2 month follow up.	Patient-rated scales at baseline and follow-up (2 months)	Physician initial communication style was positively correlated with medication adherence (p <0.05) as was the physician's follow up style (p<0.001). Collaborative communication style predicted follow up communication style (p<0.02), more positive initial beliefs about the antidepressant by the client (*p*=<0.0004) and better medication adherence (p<0.03).
**Madsen et. al (2009) [34]**	50 veterab outpatients with major depressive disorder	Not stated (longitudinal prospective design)	Patient self-report based on the Anti-depressant Questionnaire and Proportion of Days Adherent calculated	Physician Assessment Questionnaire, patient-rated	Associations between the 6-item PAQ collaboration subscale and adherence at 3, 6, 9 and 12-week postbaseline, were not statistically significant (all ps> .10)
**Mahone, I. et al (2008) [35]**	85 patients community mental health centre	Cross-sectional correlational	Patient self-report using the Schizophrenia Outcomes Module Medication (SCHIZOM) Use questionnaire.	Participation preferences and actual participation were measured using the Control preferences scale (CPS)	Neither client pariticpation, SDM oreference nor preference-participation agreement was found to be be associated with better rates of medication adherence for the past month or past 6 months.
**Hamann (2006) [36]**	107 psychiatric state hospital inpateints with a diagnosis of schizoprhenia (ICD-10)	Cluster-randomized control trial	Composite measure of adherence consisting of the MARS questionnaire (patient-rated ,derived from DAI and Maedication Adherence Questionnaire (MAQ)), doctor rating of compliance and plasma levels of antipsychotics at 6 month and 18 month follow up .	Intervention: A decision aid and planning worked talk was compared to routine care	No significant effects of the intervention on adherence
**Loh (2007)[33]**	207 patients diagnosed with	Survey study and	GP- and patient rated at 6-8 weeks post	Patient participation scale.	Patient participation was positively correlated with adherence measures

**Loh (2007) [36]**	23 primary care physicians treating 405 patients with newly diagnosed depression	A cluster randomuzed control trial	Patient and provider report.	Intervention: physician training and a decision board for use during the consultation	No significant effects of the intervention on adherence
**Ludmen et al (2003) [37]**<br><br>**Von Korff et al (2003) [38}**	386 primary care patients receiving antidepressant for depression (receiving a new antidepressant prescription)	Randomised trial	Patient self-report.	Intervention: education about depression, shared decision making regarding the use of maintenance pharmacotherapy, two visits and a telephone contact with a depression prevention specialist	Intervention patients were significantly more likely to refill antidepressant medication prescriptions than usual care patients during the 1 year follow up period (adjusted odds ration intervention: control= 1.91, 95% CI (1.37, 2.65) P<0.001) Intervention patients were also more likely to receive adequate dosage of antidepressant treatment compared to usual care patients during the 1-year follow-up period (adjusted odds ratio intervention: control = 2.08, 95% CI (1.14-3.06), P<0.001)
**Sleath et. al (2003) [43]**	27 resident physicians with 6-21 patients with depression in general internal medicine and family practice	Not stated (prospective observational)	Pharmacy refill records used to calculate adherence for the 100 day period post consultation	Researchers coded for specific communication variables in audio-taped transcripts relating to discussions surrounding medication	Question asking about antidepressant adherence was related to adherence (p=0.03).
